# An intersectional analysis of maternal mortality in Sub-Saharan Africa: a human rights issue

**DOI:** 10.7189/jogh.09.010320

**Published:** 2019-06

**Authors:** Judah Batist

**Affiliations:** Faculty of Health Sciences, McMaster University, Hamilton, Ontario, Canada

Sub-Saharan Africa continues to possess the highest rates of maternal mortality across the globe. In recent years, it was estimated that almost half of all global maternal fatalities from pregnancy-related complications occurred in Sub-Saharan Africa. There has been a decline in the rates of maternal mortality in Sub-Saharan Africa since 1990. However, these rates are not uniformly distributed across the region and remain troublingly high relative to the global front [1]. Importantly, since the overwhelming majority of maternal deaths from pregnancy-related complications are a result of the inequitable and oppressive conditions plaguing that region, maternal mortality is a human rights issue. This article posits that maternal mortality in Sub-Saharan Africa is a violation of human rights through an analysis of the intersecting social determinants of gender, economics and education in the regional context. Maternal mortality must be framed and understood as a fundamental human rights issue in order to effectively ameliorate this systemic global health burden and promote human flourishing.

## SOCIAL DETERMINANTS AND INTERSECTIONALITY

There are three over-arching social determinants of maternal mortality within Sub-Saharan Africa that shape the situation as a human rights issue: gender, education and literacy, and economic factors. In order to effectively address each of these major determinants of maternal mortality in Sub-Saharan Africa, it is essential to employ the theory of intersectionality to effectively contextualize how certain positions in societies across the region intersect and build off one another to frame the issue at hand.

Gender remains a critical social determinant of maternal mortality in Sub-Saharan Africa. The striking number of women who have prematurely died during pregnancy in Sub-Saharan Africa is largely due to gender biases in the distribution of health services and marginalization of women in these societies. A high correlation between maternal mortality ratios (MMRs)- the number of maternal deaths per 100 000 live births- and gender inequalities as it relates to the accessibility of health services has been found in Angola, Botswana, Malawi, Mozambique, South Africa, Zambia and Zimbabwe [2]. Scholars have investigated the systemic gender inequalities embedded within the fabric of many Sub-Saharan African countries, including Nigeria, Tanzania, and Kenya, where male-dominated social ideals and systemic mores continue to silence and marginalize African women [3]. This gender inequality leads to societal vulnerability, where women are subjected to sexual violence and infectious diseases, such as HIV, that can lead to unwarranted pregnancies and further complicate such pregnancies, ultimately yielding higher rates of maternal mortality [2,3].

**Figure:**
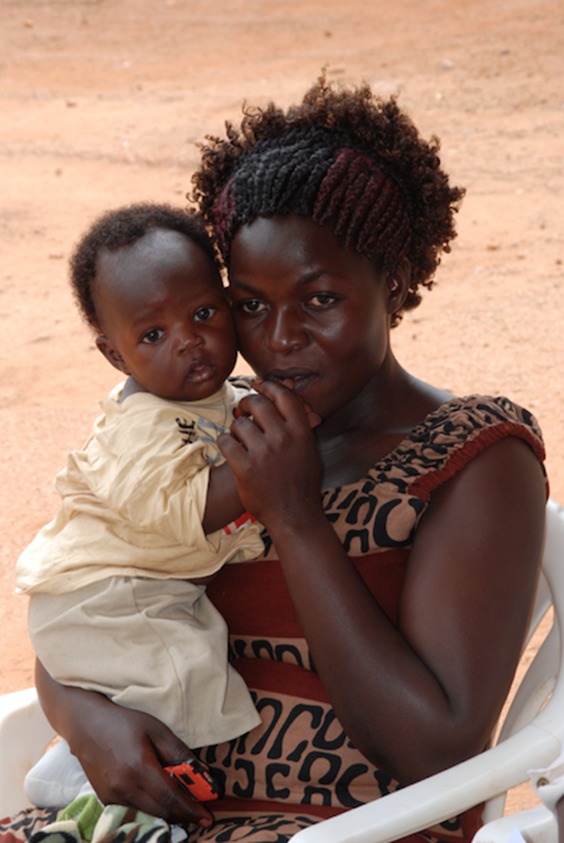
Photo: from the collection of Dr Jean Chamberlain-Froese (used with permission).

Education and literacy serves as another important social determinant of maternal mortality in Sub-Saharan Africa. Studies have found an inverse correlation between a populace’s level of education, particularly women’s education, and the country’s MMR. For example, in one analysis Mauritius demonstrated relatively high levels of education among women and therefore a relatively lower MMR [4]. Other scholars have assessed how the intersection of gender and education converge on maternal mortality within Sub-Saharan Africa. The data suggests that illiterate pregnant women are less inclined to access the necessary health services and since their illiteracy hinders their ability to be adequately informed about maternal health care options, they feel ill equipped to make autonomous decisions over their own health [2,5]. The same pattern is present in the data behind the use of contraception, where less educated women are less likely to take part in protected sexual encounters [5]. Simply put, women who are literate and have higher levels of education are able to access to the necessary information to make informed decisions about their health care and are therefore less likely to fall victim to maternal mortality.

Economic factors serve as significant determinants of maternal mortality in Sub-Saharan Africa. Researchers in the field posit a highly significant correlation between Sub-Saharan African countries Gross National Products (GNP) per capita and health status as indicated by the incidence of maternal morbidity and mortality [6]. Scholars have found that economic factors, such as per-capita government expenditure on health and Gross National Income (GNI) per capita, purveyed an inverse relationship with the nation’s MMR in Sub-Saharan Africa [4]. Simply, a nation’s economic standing has an effect on the MMR, where nations that are faring better economically have lower MMRs. An added intersecting layer to this quandary is that many women in Sub-Saharan Africa are financially dependent on their husbands, which negatively impacts women’s health-related autonomy by having to rely on their husbands’ discretions in health-related matters. Researchers have highlighted that two of the major indicators of maternal mortality are poverty and purchasing power (referring to personal expenditures) [4]. During pregnancy, women require access to adequate nutrition, health and hygiene, which are all contingent on their ability to afford and maintain such goods and services [7]. Simply, the health of pregnant women in the region is being jeopardized by insufficient access to financial resources.

## A HUMAN RIGHTS ISSUE

Research has demonstrated that the majority of women dying a maternal death fall into the category of “low-risk women” [6]. This means that the vast majority of the annual 350 000 deaths related to pregnancy and childbirth complications are preventable with low-cost, targeted interventions, policies, and services, all of which are understood as truisms in the medical profession and health systems for decades [8]. The avoidable nature of most maternal deaths related to pregnancy and childbirth in Sub-Saharan Africa brings to focus the sobering realities of a disadvantaged developing world where there dissonance between developed and developing countries has long been cited as the “largest discrepancy of all public-health statistics” [9]. In order to elevate these injustices from their statistics, maternal mortality must be understood as a basic human rights issue disproportionately impacting the developing world. Framing the issue of maternal mortality according to rights-based terminology makes palpable the underlying connections between poverty, discrimination, equality and health, as well as pushes the agenda of protecting and fulfilling our basic human right to health to the forefront of global discourse [8].

A human rights-based approach to maternal mortality highlights how these deaths trigger and aggravate cycles of intergenerational oppression and marginalization. The death of a mother tremendously disadvantages her children’s future. The research demonstrates that, without mothers, children, and specifically female children, remain at greater risk for dropping out of school, becoming malnourished and simply not surviving [8]. Losing women and mothers to preventable pregnancy and childbirth complications is therefore part of a larger systemic malfunction, where socioeconomic immobility fuels continued experiences of female oppression. The goal of a human rights-based approach to maternal mortality and morbidity is to empower women by putting pressure on those in positions of power to influence political change. This means providing women with the opportunity “to exercise their right to participate in decision-making processes, including those affecting their sexual and reproductive health, family planning, contraception, pregnancy, childbirth and in addressing unsafe abortion” [8].

Global accountability, equality, non-discrimination and meaningful participation are central human rights principles raised and are the constructs most threatened by the burden of maternal mortality. The failings of those in positions of power to fulfill their duties to protect and promote women’s flourishing are unearthed by shifting the discourse to focus on female empowerment and their right to self-determination. Thus, only when the discussion revolves around a violation of fundamental human rights will remedial measures be put in place.

## CRITIQUE OF EXISTING DIRECTIVES AND INSIGHTS FOR ALLEVIATING BARRIERS

A review of the combative measures to alleviate maternal deaths centralizes on improving the quality and accessibility of health care and related personnel. Early detection and treatment of anemia in pregnancy, active management of the third stage and use of prophylactic oxytocics, as well as rapid recognition of warning signs are required measures for reducing maternal, neonatal and childhood mortality. Blood transfusion services and proper management of obstetric emergencies are also considered neglected health care components in many sub-Saharan countries [10].

Researchers have indicated that the maternal mortality rate of a country directly correlates to the availability of skilled delivery personnel, life expectancy, national economic wealth and health expenditure per capita [6]. Based on these findings, scholars suggest that structural arrangements be made in order to train more skilled health personnel to take care of maternal health problems. Considering the shortage of physicians in the region and the relatively high cost of training physicians, several scholars recommend training middle-level health personnel as an affordable alternative for handling emergency obstetrical cases. Given the poor economic status of sub-Saharan African countries, educating middle-level health workers is a more financially feasible project than training gynecologists to provide antenatal and postnatal services [4,6,8,9]. However, in spite of the many efforts made in sub-Saharan countries to reduce the MMR in recent decades, the progress has been minimal. This has largely to do with the fact that isolating maternal mortality to a singular relationship with health care systems and/or biomedical related-variables neglects the importance of education, gender and economic variables in their abilities to stagnate the potential for improvement [8].

Moving forward, two conclusions can be drawn from this analysis. First, it is impossible to ameliorate the barriers to maternal health without properly outlining what those barriers are. Focusing solely on the health care system and/or biomedical related-variables fails to account for the systemic determinants of maternal mortality in Sub-Saharan Africa. This analysis has demonstrated that the intersections of gender, education and economic factors create tangible barriers to maternal health that, without proper attention, will continue to remain unchallenged. This lends itself to the second conclusion, which is that maternal mortality must be framed and understood as a human rights issue. In order for the aforementioned determinants of gender, education and economics to receive their due diligence, contextualizing maternal mortality as a basic human rights issue politicizes these underlying factors and forces those in positions of power to consider these antecedent conditions as part of their political agendas. This politicization should not be isolated to the nation-states themselves, but should additionally manifest in the platforms and policies outlined by NGOs and other international health organizations. It is essential for international organizations and political actors to take initiative when it comes to developing novel research on the implications that gender, education, and economics have on maternal health in Sub-Saharan Africa. This will not only re-define maternal mortality as a human rights issue in health discourse across the globe, but will also aid in driving policy and interventions that center on these social determinants of health.
